# Shared and distinct patterns of dynamical degree centrality in bipolar disorder across different mood states

**DOI:** 10.3389/fpsyt.2022.941073

**Published:** 2022-07-27

**Authors:** Fuping Sun, Zhening Liu, Jun Yang, Zebin Fan, Chang Xi, Peng Cheng, Zhong He, Jie Yang

**Affiliations:** ^1^Department of Psychiatry, National Clinical Research Center for Mental Disorders, The Second Xiangya Hospital of Central South University, Changsha, China; ^2^Department of Radiology, The Second Xiangya Hospital of Central South University, Changsha, China

**Keywords:** bipolar disorder, dynamical degree centrality, inferior parietal lobule, precuneus, posterior cingulate cortex, middle occipital gyrus

## Abstract

**Background:**

Previous studies have probed the brain static activity pattern in bipolar disorder across different states. However, human intrinsic brain activity is time-varying and dynamic. There is a lack of knowledge about the brain dynamical pattern in bipolar disorder across different mood states.

**Methods:**

This study used the dynamical degree centrality (dDC) to investigate the resting-state whole-brain dynamical pattern voxel-wise in a total of 62 bipolar disorder [28 bipolar depression (BD), 13 bipolar mania (BM), 21 bipolar euthymia (BE)], and 30 healthy controls (HCs). One-way analysis of variance (ANOVA) was applied to explore the omnibus differences of the dDC pattern across all groups, and Pearson’s correlation analysis was used to evaluate the relationship between the dDC variability in detected regions with clinical symptom severity.

**Results:**

One-way ANOVA analysis showed the omnibus differences in the left inferior parietal lobule/middle occipital gyrus (IPL/MOG) and right precuneus/posterior cingulate cortex (PCUN/PCC) across all groups. The *post hoc* analysis revealed that BD showed decreased dDC in the IPL/MOG compared with all other groups, and both BD and BM exhibited decreased dDC in the PCUN/PCC compared with BE and HCs. Furthermore, correlation analysis showed that the dDC variability of the IPL/MOG and PCUN/PCC negatively correlated with the depression symptom levels in all patients with bipolar disorder.

**Conclusion:**

This study demonstrated the distinct and shared brain dynamical pattern of the depressive, manic, and euthymia states. Our findings provide new insights into the pathophysiology of bipolar disorder across different mood states from the dynamical brain network pattern perspective.

## Introduction

Bipolar disorder (BD) is a mood disorder with alternating periods of depression (hypo) mania [bipolar mania (BM)], and euthymic mood states [bipolar euthymia (BE)] (1). It affects > 1% of the global population and is a leading cause of disability worldwide (2). Previous studies have reported the abnormal structural, activation, and functional connectivity underlying bipolar disorders (3–6). However, limited studies have considered the various mood episodes in bipolar disorders. Therefore, further research is needed to investigate the pathophysiological mechanisms of bipolar disorder across different mood states (7, 8).

Previous studies have explored the distinctive neural mechanism underlying different mood states of bipolar disorder. For example, Martino et al. have reported the sensorimotor and default-mode networks (DMNs) showed opposite variation patterns in BD and BM (9). Russo et al. have documented that BD showed the altered regional homogeneity in the primary sensorimotor cortex, and BM showed altered regional homogeneity and degree centrality (DC) in the medial prefrontal cortex (10). These findings may indicate that the functional connectivity imbalance pattern between the sensorimotor and DMN is related to the clinical mood state of bipolar disorder. Meanwhile, a prior study has reported that both BD and BM showed higher activation in the right insula, right putamen, and left lateral prefrontal cortex when inhibiting sad faces compared with BE and healthy controls (HCs) (11). In addition, compared with HCs, both BD and BM showed reduced activation in the dorsolateral prefrontal cortex during an N-back working memory task, but there were no differences manifested compared with BE (12). These findings indicated that they have shared and distinct brain patterns among the different mood states of bipolar disorder.

It has been reported that the human brain is a highly dynamic and time-varying system and maintains a dynamic balance to ensure effective communication between various brain regions (13–15). The analysis methods of looking into the brain dynamical pattern have been adopted for investigating neural mechanisms of mental disorders (16–18). For example, Fu et al. have reported the dynamical pattern in patients with schizophrenia by using the dynamical low-frequency fluctuation amplitude and dynamical functional connectivity (16), and our prior studies also have investigated the differences between BD and unipolar depression by using the dynamical fractional amplitude of low-frequency fluctuations and dynamical regional homogeneity, respectively (19, 20). However, to the best of our knowledge, no study has investigated the brain dynamical pattern in bipolar disorder across three different mood states. Thus, this study aimed to fill this blank.

In this study, we used the voxel-wise dynamic degree centrality DC (dDC) approach to explore the dynamical pattern of the resting-state whole-brain functional connectome in bipolar disorder across three states. DC is an index of the total weight of connections for a given node (15) and has been widely used in psychiatric disorders as an analytic measurement to reveal the core-hub architecture of brain networks (21–23). The dDC approach adds the sliding-window step to measure the time-varying features of the DC maps (24). By adopting this method, we first hypothesized that the dynamical pattern of the whole-brain functional connectome will be distinctive across three mood states (i.e., mania, depression, euthymia), and it will be strongly related to the severity of the clinical symptoms. We further hypothesized that the distinctive dDC value across three states would tend to be distributed in the DMN and sensorimotor areas that have been repeatedly reported in previous studies. By studying the differences in bipolar disorder across different mood states in terms of dDC, we can have a more comprehensive understanding of the bipolar disorder.

## Materials and methods

### Participants

A total of 77 patients with bipolar disorder were enrolled in this study, including 32 patients with BD, 20 patients with BM, and 25 patients with BE. All patients were recruited from the inpatient or outpatient department of the Second Xiangya Hospital, Central South University. To reduce the heterogeneous impact of different typing patients with bipolar disorder, our study only included patients with type I bipolar disorder (25). They were diagnosed as bipolar disorder type I (including the BD, BM, or BE) using the Structured Clinical Interview for DSM-IV Axis I Disorders, Patient Edition (SCID-I/P) (26). The clinical symptoms were comprehensively assessed by two experienced psychiatrists using the Hamilton Depression Scale (HAMD) (27), Young Mania Rating Scale (YMRS) (28), Hamilton Anxiety Rating Scale (HAMA) (29), and Brief Psychiatric Rating Scale (BPRS) (30), respectively. HAMD is the most commonly used scale to clinically assess depression status and its severity. HAMA is mainly used to assess the severity of anxiety symptoms in patients. YMRS is mainly used to assess manic symptoms and their severity. BPRS is mainly used to assess the severity of the patient’s psychiatric symptoms. The inclusion criteria were the HAMD score ≥ 17 and the YMRS score < 6 for BD; the YMRS score ≥ 12 and the HAMD score < 8 for BM; the HAMD score < 8 and the YMRS score < 6 for BE (31, 32).

A total of 35 HCs were recruited from the local universities and communities through advertisement and using the Structured Clinical Interview for DSM-IV, Non-patient Edition (SCID-I/NP). To better understand our sample, all participants were assessed by using the Wechsler Adult Intelligence Scale (WAIS) (33), including WAIS-Knows and WAIS-Digit symbol.

All participants were excluded if they had any of the following: (1) < 18 years old or > 45 years old; (2) previous neurological diseases or serious physical illness; (3) previous alcohol or other psychoactive substance abuse; (4) previous electroconvulsive therapy; and (5) had taken benzodiazepines within 24 h before scanning or any other contraindications to MRI.

This study was conducted in accordance with the ethical guidelines of the medical ethics committee of the Second Xiangya Hospital, Central South University, and in strict accordance with the Declaration of Helsinki. Participants were informed and agreed to participate in the study and were free to withdraw from the research at any time.

### Data acquisition and preprocessing

All MRI data were collected as soon as possible after the patient’s first visit to the clinic using a 3.0 Tesla Philips Gyrosan Achieva (Amsterdam, The Netherlands) scanner. In the scanning, all participants were explicitly instructed to keep their eyes closed and stay awake. A gradient-echo echo-planar imaging sequence was used with the following parameters: axial slice = 36, matrix = 64 × 64, repeat time = 2,000 ms, echo time = 30 ms, field of view = 240 mm × 240 mm, flip angle = 90°, slice thickness = 4 mm, scanning interval = 0 mm, in a total of 250 total volumes.

Data preprocessing was carried out using DPABI^1^ (34). The first 10 volumes were removed to allow magnetization balance and adaptation to the environment (35). The remaining 240 functional scans were done with the following analyses: all participants had < 3 mm maximum displacement in x, y, or z and less than 3° of angular rotation about each axis, spatial normalization of the Montreal Neurological Institute (MNI), resampled to the voxel size of 3 × 3 × 3 mm^3^ then the BOLD signal of each voxel was detrended to abandon linear trends and passed through a bandpass filter (0.01–0.08 Hz). We also employed the scrubbing step by removing outlier volumes with frame-wise displacement (FD) > 0.5 mm. Finally, interference covariates were regressed out from the BOLD signals, including six head motions and their temporal first derivatives (36), global mean signals, white matter signals, and cerebrospinal fluid signals.

A total of 20 participants were excluded due to their excessive head movement or inability to cooperate during fMRI scanning. After data quality control, 92 subjects (including 28 patients with BD, 13 patients with BM, 21 patients with BE, and 30 patients with HCs) participated in the following analyses.

### Temporal variability of the dynamical degree centrality

**FIGURE 1:**
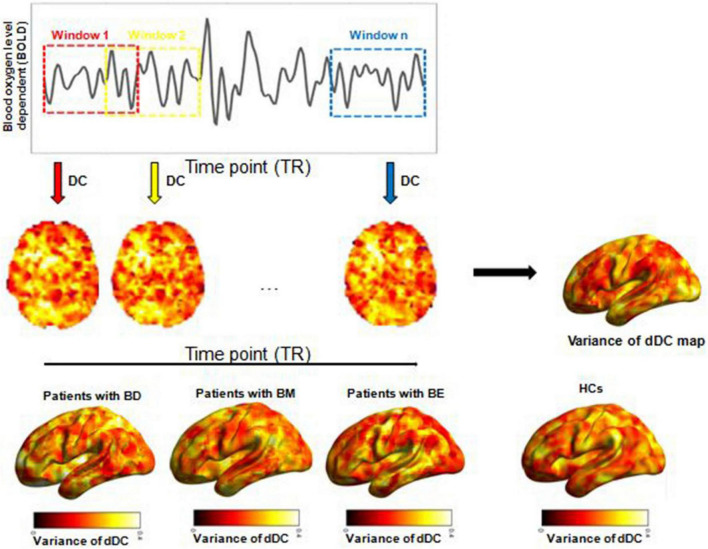
Illustration of analysis steps. dDC, dynamical degree centrality; BD, bipolar depression; BM, bipolar mania; BE, bipolar euthymia; HCs, healthy controls.

As mentioned earlier, DC is a widely used method to describe intrinsic brain connectivity at a global level (37). In this study, by using the Dynamic Brain Connectome (DynamicBC) toolbox, the temporal variability of voxel-wise dDC was calculated according to the sliding-window approach. According to the recommendations of the previous studies (38), we used a window length of 50 TRs (100 s) to calculate the temporal variability of dDC. We obtained a dDC map for each sliding window and then computed the coefficient of variation across all sliding windows to explore the dDC variability of the four groups. Finally, the dDC map was smoothed with full-width at half maximum = 6 mm. The analytical steps of this study are illustrated in Figure 1.

### Validation analyses

To validate our main findings, we reperformed the analysis by using a sliding-window length of 70 TRs (140 s).

### Statistical analysis

The demographic and clinical characteristics of the four groups have been analyzed using SPSS 21.0. Differences in age, education, illness duration, age of onset, manic episodes times, depressive episodes times, mean FD, WAIS–Knows, WAIS-Digit symbol, HAMD, YMRS, HAMA, BPRS, and chlorpromazine (CPZ) equivalents (39) were analyzed with a one-way analysis of variance (ANOVA). A chi-square test was used to calculate the differences in gender across all groups.

The dDC variability among the four groups was performed using Statistic Parameter Mapping 8 software.^2^ One-way ANOVA was carried out to compare the dDC variability among the due groups voxel-wise with age, gender, and education years as nuisance covariates. Then, by applying the significant voxels that survived in one-way ANOVA analysis as the mask, the *post hoc t*-tests were performed between any two groups. The threshold was set at voxel-level *p*_voxel_ < 0.005 and cluster size > 60 (AlphaSim corrected, *p*_cluster_ < 0.05).

Finally, Pearson’s correlation analysis was used to evaluate the relationship between the altered dDC variability with the HAMD score and YMRS score.

## Results

### Demographic and clinical characteristics

No significant differences were detected for age [*F*_(3,91)_ = 0.710, *p* = 0.548], gender (χ*^2^* = 0.174, *p* = 0.677), education [*F*_(3,91)_ = 2.440, *p* = 0.070], mean FD [*F*_(3,91)_ = 1.517, *p* = 0.216], and WAIS-knows [*F*_(3,91)_ = 1.216, *p* = 0.309] in all groups. However, WAIS-Digit symbol score of the three patient groups was lower than that of HCs [*F*_(3,91)_ = 11.507, *p* < 0.001]. Three patient groups were matched with illness duration [*F*_(3,91)_ = 1.374, *p* = 0.261], age of onset [*F*_(3,91)_ = 0.116, *p* = 0.891], manic episodes times [*F*_(3,91)_ = 0.576, *p* = 0.565], and depressive episodes times [*F*_(3,91)_ = 1.153, *p* = 0.323]. In addition, BD showed a higher score of HAMD [*F*_(3,91)_ = 122.500, *p* < 0.001], HAMA [*F*_(3,91)_ = 33.129, *p* < 0.001], BPRS [*F*_(3,91)_ = 13.874, *p* < 0.001] than that of BM and BE, while BM showed a higher score of YMRS [*F*_(3,__91)_ = 140.063, *p* < 0.001] than that of BD and BE. Moreover, significant differences were found for CPZ equivalents [*F*_(3,91)_ = 7.413, *p* = 0.001] in three patient groups, and BE took more antipsychotics than that of BD (*t* = 2.80, *p* = 0.014) and BM (*t* = 1.917, *p* = 0.073). Detailed results are shown in Table 1 and Supplementary Table 1.

**TABLE 1:** Cohort demographics and clinical characteristics.

Characteristics	BD (*n* = 28)	BM (*n* = 13)	BE (*n* = 21)	HCs (*n* = 30)	*F/*χ ^2^	*P*
Age (years)	26.50 ± 6.76	27.69 ± 7.34	26.95 ± 6.25	24.93 ± 6.17	0.710	0.548^b^
Gender (M/F)	12/16	5/8	11/10	15/15	0.174	0.677^a^
Education (years)	13.04 ± 3.05	11.31 ± 3.01	13.98 ± 2.60	12.73 ± 2.69	2.440	0.070^b^
Illness duration (months)	52.83 ± 53.16	78.38 ± 91.57	42.86 ± 40.47	N/A	1.374	0.261^b^
Manic episodes (times)	2.32 ± 2.93	3.38 ± 3.20	2.67 ± 2.82	N/A	0.576	0.565^b^
Depressive episodes (times)	3.54 ± 3.17	2.08 ± 2.33	4.05 ± 4.92	N/A	1.153	0.323^ b^
Age of onset (years)	21.43 ± 4.29	21.75 ± 6.59	22.15 ± 5.25	N/A	0.116	0.891^b^
WAIS-Knows	19.64 ± 4.74	18.05 ± 4.98	20.37 ± 4.38	20.69 ± 4.33	1.216	0.309^b^
WAIS-Digit symbol	65.29 ± 18.29	62.83 ± 14.87	73.33 ± 13.39	84.97 ± 10.31	11.507	<0.001^b^
CPZ-equivalents (mg)	13.10 ± 51.39	50.00 ± 99.54	149.89 ± 188.18	N/A	7.413	0.001^b^
Mean FD	0.12 ± 0.36	0.12 ± 0.03	0.14 ± 0.59	0.15 ± 0.10	1.517	0.216^b^

^a^p-value for chi-square test.

^b^p-values for one-way ANOVA. Values are presented by mean ± standard deviation. BD, bipolar depression; BM, bipolar mania; BE, bipolar euthymia; HCs, healthy controls; WAIS, Wechsler Intelligence Scale; CPZ, chlorpromazine; FD, frame-wise displacement; N/A, not available.

### Temporal variability of the dynamical degree centrality

**FIGURE 2:**
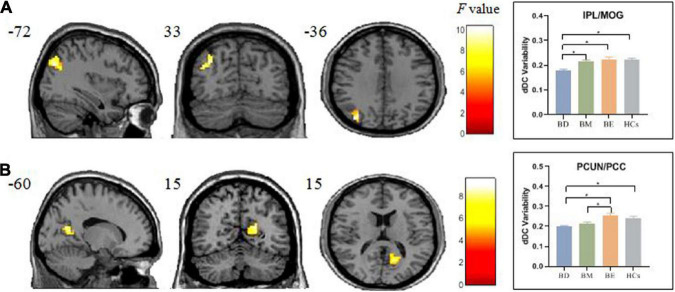
Brain regions showed significant omnibus differences of the dDC variability among four groups. **(A)** The BD group showed a decreased dDC variability compared with all other groups in IPL/MOG. **(B)** Both the BD and BM groups exhibited decreased dDC compared with BE in PCUN/PCC. *p < 0.05. dDC, dynamical degree centrality; BD, bipolar depression; BM, bipolar mania; BE, bipolar euthymia; HCs, healthy controls; IPL/MOG, inferior parietal lobule/middle occipital gyrus; PCUN/PCC, precuneus/posterior cingulate cortex.

One-way ANCOVA showed significant dDC variability differences among the four groups in left inferior parietal lobule/middle occipital gyrus (IPL/MOG; MNI [x = −36, y = −72, z = 33]; *F*_(3,91)_ = 9.58; see Figure 2 and Table 2) and right precuneus/posterior cingulate (PCUN/PCC; MNI [x = 15, y = −60, z = 15]; *F*_3_,_91_ = 9.04; see Figure 2 and Table 2). The *post hoc t*-tests revealed that BD showed decreased dDC in the IPL/MOG compared with BM (*t* = −3.76), BE (*t* = −4.66), and HCs (*t* = −4.77). The *post hoc t*-tests revealed that BD showed decreased dDC in the PCUN/PCC compared with BE (*t* = −4.26) and HCs (*t* = 4.21), and BM showed decreased dDC in the PCUN/PCC compared with BE (*t* = −3.85). In addition, we did not observe any significant correlations between the dDC variability of areas with omnibus differences and mean FD in all participants (see Supplementary Figure 1).

**TABLE 2:** Brain regions with significant dDC difference among the four groups.

One-way ANOVA						*F*	*Post hoc* analysis	
Brain region	MNI			BA	Voxels		Comparisons	*T*
	*X*	*Y*	*Z*					
IPL/MOG	−36	−72	33	19	62	9.58	BD < BM BD < BE BD < HCs	−3.76 −4.66 −4.77
PCUN/PCC	15	−60	15	30	63	9.04	BD < BE BD < HCs BM < BE	−4.26 −4.21 −3.85

dDC, the dynamical degree centrality; BD, bipolar depression; BM, bipolar mania; BE, bipolar euthymia; HCs, healthy controls; MNI, Montreal Neurological Institute; BA, Brodmann area; IPL/MOG, inferior parietal lobule/middle occipital gyrus; PCUN/PCC, precuneus/posterior cingulate cortex.

### Validation results

We also observed the omnibus dDC differences in IPL/MOG and PCUN/PCC using 70 TRs. The results were essentially in agreement with that of 50 TRs, and the details are summarized in Supplementary Table 2.

### Correlation analysis

**FIGURE 3:**
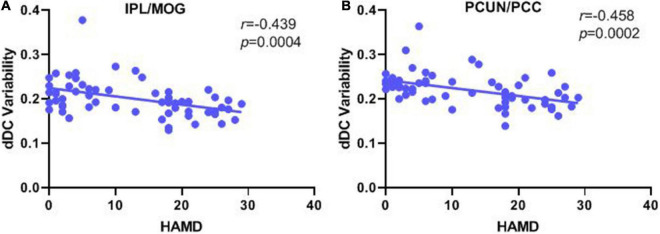
Correlation analysis of the dDC variability with manic/depressive symptoms in all patients with bipolar disorder. **(A)** Pearson’s correlation between the altered dDC variability in the IPL/MOG with HAMD score. **(B)** Pearson’s correlation between the altered dDC variability in PCUN/PCC with the HAMD score. dDC, dynamical degree centrality; BD, bipolar depression; BM, bipolar mania; BE, bipolar euthymia; HCs, healthy controls; IPL/MOG, inferior parietal lobule/middle occipital gyrus; PCUN/PCC, precuneus/posterior cingulate cortex.

Correlation analyses revealed significant negative correlations between the HAMD score and the dDC variability of the IPL/MOG (*r* = −0.439, *p* < 0.001) and PCUN/PCC (*r* = −0.458, *p* < 0.001), but no significant correlation was found between the YMRS score and the dDC variability of the IPL/MOG (*r* = 0.192, *p* = 0.138) and PCUN/PCC (*r* = 0.177, *p* = 0.172) (see Figure 3 and Table 3).

**TABLE 3:** Correlation analysis of dDC variability with manic/depressive symptoms in all patients with bipolar disorder.

Variable		IPL/MOG	PCUN/PCC
HAMD	*r*	-0.439	**−**0.458
	*p*	0.000**	0.000**
YMRS	*r*	0.192	0.177
	*p*	0.138	0.172

**p < 0.001. dDC, the dynamical degree centrality; HAMD, 17-item Hamilton Depression Rating Scale; YMRS, Young Mania Rating Scale; IPL/MOG, inferior parietal lobule/middle occipital gyrus; PCUN/PCC, precuneus/posterior cingulate cortex.

## Discussion

To the best of our knowledge, this is the first study to investigate the dDC variability of the whole-brain functional connectome at the voxel-wise across all diagnostic groups. We found significant dDC differences among all groups in the IPL/MOG and PCUN/PCC. More specifically, the BD group showed a decreased dDC in the IPL/MOG compared with all other groups. Both the BD and BM groups exhibited decreased dDC in PCUN/PCC compared with BE. Furthermore, correlation analysis showed that dDC variability of the IPL/MOG and PCUN/PCC negatively correlated with scores of depressive symptoms in all patients.

We observed decreased dDC variability of the IPL/MOG in BD compared with other groups, and the dDC variability negatively correlated with the HAMD scores. The IPL/MOG involves emotion regulation, reaction inhibition, and self-circulation processing (40). Qiu et al. have reported that both patients with BD and patients with major depressive disorder showed abnormal fractional ALFF in the IPL/MOG compared with HCs (41). Zhang et al. have documented those patients with BE and BD manifested disrupted functional connectivity in IPL/MOG during resting-state (42). Furthermore, consistent with this study, Luo et al. have adopted the dynamical functional connectivity and observed that both patients with BD and patients with major depressive disorder displayed reduced dynamical functional connectivity between the IPL/MOG and precuneus (43). These studies may suggest that deficits of the static or dynamical regional activity or distal functional connectivity in the IPL/MOG are more pronounced in the depressive state. Accordingly, we speculate that the IPL/MOG would be a potential biomarker that distinguishes BD from BM and BE.

Compared with BE, both BD and BM exhibited decreased dDC in the PCUN/PCC, while no significant difference was found for BD and BM. Consistent with our results, a meta-analysis based on large samples reported that both BD and BM presented decreased connectivity within the DMN, but BE showed increased connectivity (44). The PCUN/PCC is a crucial component in the DMN and acts as an intermediate hub with other networks (45). Previous studies have documented patients with bipolar disorder manifested imbalanced functional connectivity between DMN and salience network (46). It was reported that BD showed decreased functional connectivity between the perigenual anterior cingulate cortex and anterior cingulate cortex, and BM showed decreased functional connectivity between the perigenual anterior cingulate cortex and PCUN/PCC.

We also observed the abnormal dDC in the PCUN/PCC correlated with depressive symptoms. Zhong et al. have documented that patients with BD showed intrinsic activity abnormalities under specific frequency bands in the PCUN/PCC and MOG (47). A prior study has reported that patients with BM showed decreased connectivity within the PCUN/PCC, and it correlated with clinical severity scores (48). Combining with our findings of the shared decreased dDC in the PCUN/PCC between the BD and BM groups, we speculate that this area may have important implications to be a potential intervention target for bipolar disorder during active phases (i.e., BD and BM).

It should be noted that this study has some limitations. First, our small sample size, especially the BM group (the sample size is 13), which may limit the statistic power and easily cause the type I and II errors, and the results should be considered preliminary. In addition, this is a cross-sectional study that can only observe the current time measurements of the brain dynamical connectivity pattern, and we did not collect the longitudinal data of patients with bipolar disorder in different mood states. Therefore, further longitudinal studies with larger samples are needed to verify our results, and any generalization of the findings of this study needs to be carefully made. Finally, considering the drugs taken by the patients at the time of enrollment, we cannot ignore the possible impact of drugs on our results.

## Conclusion

Our study investigated the brain dynamical pattern in bipolar disorder across different mood states by using the dDC variability index. We observed the detected dDC abnormalities in IPL/MOG were unique to BD, and the dDC abnormalities PCUN/PCC both manifested in BD and BM. It may indicate that there are common and different patterns of the dynamic brain connectome pattern of bipolar disorder in different states. Our findings provide an insight into the resting-state global brain network dynamics in bipolar disorder.

## Data availability statement

The raw data supporting the conclusions of this article will be made available by the authors, without undue reservation.

## Ethics statement

The studies involving human participants were reviewed and approved by the medical Ethics Committee of the Second Xiangya Hospital, Central South University. The patients/participants provided their written informed consent to participate in this study.

## Author contributions

ZL, ZH, and JiY designed the study. FS, JuY, ZF, CX, and PC acquired the data. FS, JuY, and ZF analyzed the data. FS and JiY wrote the article. All authors reviewed the article and authors approved the final version to be published and can certify that no other individuals not listed as authors have made substantial contributions to the article.

## Conflict of interest

The authors declare that the research was conducted in the absence of any commercial or financial relationships that could be construed as a potential conflict of interest.

## Publisher’s note

All claims expressed in this article are solely those of the authors and do not necessarily represent those of their affiliated organizations, or those of the publisher, the editors and the reviewers. Any product that may be evaluated in this article, or claim that may be made by its manufacturer, is not guaranteed or endorsed by the publisher.

## Footnotes

1
http://rfmri.org/dpabi

2
www.fil.ion.ucl.ac.uk/spm

## References

[1] Alonso J Petukhova M Vilagut G Chatterji S Heeringa S Ustun TB Days out of role due to common physical and mental conditions: results from the WHO world mental health surveys. *Mol Psychiatry.* (2011) 16:1234–46. 10.1038/mp.2010.101 20938433 PMC3223313

[2] Grande I Berk M Birmaher B Vieta E. Bipolar disorder. *Lancet.* (2016) 387:1561–72. 10.1016/S0140-6736(15)00241-X 26388529

[3] Skatun KC Kaufmann T Tonnesen S Biele G Melle I Agartz I Global brain connectivity alterations in patients with schizophrenia and bipolar spectrum disorders. *J Psychiatry Neurosci.* (2016) 41:331–41. 10.1503/jpn.150159 26854755 PMC5008922

[4] Zhou Q Womer FY Kong L Wu F Jiang X Zhou Y Trait-related cortical-subcortical dissociation in bipolar disorder: analysis of network degree centrality. *J Clin Psychiatry.* (2017) 78:584–91. 10.4088/JCP.15m10091 28002659

[5] Wolfers T Rokicki J Alnaes D Berthet P Agartz I Kia SM Replicating extensive brain structural heterogeneity in individuals with schizophrenia and bipolar disorder. *Hum Brain Mapp.* (2021) 42:2546–55. 10.1002/hbm.25386 33638594 PMC8090780

[6] Serafini G Pompili M Borgwardt S Houenou J Geoffroy PA Jardri R Brain changes in early-onset bipolar and unipolar depressive disorders: a systematic review in children and adolescents. *Eur Child Adolesc Psychiatry.* (2014) 23:1023–41. 10.1007/s00787-014-0614-z 25212880

[7] Harrison PJ Geddes JR Tunbridge EM. The emerging neurobiology of bipolar disorder. *Focus (Am Psychiatr Publ).* (2019) 17:284–93. 10.1176/appi.focus.17309 32015720 PMC6996057

[8] Baldessarini RJ Innamorati M Erbuto D Serafini G Fiorillo A Amore M Differential associations of affective temperaments and diagnosis of major affective disorders with suicidal behavior. *J Affect Disord.* (2017) 210:19–21. 10.1016/j.jad.2016.12.003 27992854

[9] Martino M Magioncalda P Huang Z Conio B Piaggio N Duncan NW Contrasting variability patterns in the default mode and sensorimotor networks balance in bipolar depression and mania. *Proc Natl Acad Sci USA.* (2016) 113:4824–9. 10.1073/pnas.1517558113 27071087 PMC4855585

[10] Russo D Martino M Magioncalda P Inglese M Amore M Northoff G. Opposing changes in the functional architecture of large-scale networks in bipolar mania and depression. *Schizophr Bull.* (2020) 46:971–80. 10.1093/schbul/sbaa004 32047938 PMC7342167

[11] Hummer TA Hulvershorn LA Karne HS Gunn AD Wang Y Anand A. Emotional response inhibition in bipolar disorder: a functional magnetic resonance imaging study of trait– and state-related abnormalities. *Biol Psychiatry.* (2013) 73:136–43. 10.1016/j.biopsych.2012.06.036 22871393 PMC5821068

[12] Pomarol-Clotet E Alonso-Lana S Moro N Sarro S Bonnin MC Goikolea JM Brain functional changes across the different phases of bipolar disorder. *Br J Psychiatry.* (2015) 206:136–44. 10.1192/bjp.bp.114.152033 25497296

[13] Faghiri A Stephen JM Wang YP Wilson TW Calhoun VD. Changing brain connectivity dynamics: from early childhood to adulthood. *Hum Brain Mapp.* (2018) 39:1108–17. 10.1002/hbm.23896 29205692 PMC5807176

[14] Allen EA Damaraju E Plis SM Erhardt EB Eichele T Calhoun VD. Tracking whole-brain connectivity dynamics in the resting state. *Cereb Cortex.* (2014) 24:663–76. 10.1093/cercor/bhs352 23146964 PMC3920766

[15] Marusak HA Calhoun VD Brown S Crespo LM Sala-Hamrick K Gotlib IH Dynamic functional connectivity of neurocognitive networks in children. *Hum Brain Mapp.* (2017) 38:97–108. 10.1002/hbm.23346 27534733 PMC5796541

[16] Fu Z Tu Y Di X Du Y Pearlson GD Turner JA Characterizing dynamic amplitude of low-frequency fluctuation and its relationship with dynamic functional connectivity: an application to schizophrenia. *Neuroimage.* (2018) 180:619–31. 10.1016/j.neuroimage.2017.09.035 28939432 PMC5860934

[17] Rashid B Damaraju E Pearlson GD Calhoun VD. Dynamic connectivity states estimated from resting fMRI Identify differences among schizophrenia, bipolar disorder, and healthy control subjects. *Front Hum Neurosci.* (2014) 8:897. 10.3389/fnhum.2014.00897 25426048 PMC4224100

[18] Qiu L Xia M Cheng B Yuan L Kuang W Bi F Abnormal dynamic functional connectivity of amygdalar subregions in untreated patients with first-episode major depressive disorder. *J Psychiatry Neurosci.* (2018) 43:262–72. 29947609 10.1503/jpn.170112 PMC6019355

[19] Sun F Liu Z Fan Z Zuo J Xi C Yang J. Dynamical regional activity in putamen distinguishes bipolar type I depression and unipolar depression. *J Affect Disord.* (2021) 297:94–101. 10.1016/j.jad.2021.10.021 34678402

[20] Sun F Liu Z Yang J Fan Z Yang J. Differential dynamical pattern of regional homogeneity in bipolar and unipolar depression: a preliminary resting-state fMRI study. *Front Psychiatry.* (2021) 12:764932. 10.3389/fpsyt.2021.764932 34966303 PMC8710770

[21] Wang H Zhang B Zeng B Tang Y Zhang T Zhao S Association between catechol-O-methyltransferase genetic variation and functional connectivity in patients with first-episode schizophrenia. *Schizophr Res.* (2018) 199:214–20. 10.1016/j.schres.2018.04.023 29730044

[22] Zhang J Wang J Wu Q Kuang W Huang X He Y Disrupted brain connectivity networks in drug-naive, first-episode major depressive disorder. *Biol Psychiatry.* (2011) 70:334–42. 10.1016/j.biopsych.2011.05.018 21791259

[23] Deng W Zhang B Zou W Zhang X Cheng X Guan L Abnormal degree centrality associated with cognitive dysfunctions in early bipolar disorder. *Front Psychiatry.* (2019) 10:140. 10.3389/fpsyt.2019.00140 30949078 PMC6435527

[24] Wang Y Jiang Y Su W Xu L Wei Y Tang Y Temporal dynamics in degree centrality of brain functional connectome in first-episode schizophrenia with different short-term treatment responses: a longitudinal study. *Neuropsychiatr Dis Treat.* (2021) 17:1505–16. 10.2147/NDT.S305117 34079256 PMC8166279

[25] Ha TH Her JY Kim JH Chang JS Cho HS Ha K. Similarities and differences of white matter connectivity and water diffusivity in bipolar I and II disorder. *Neurosci Lett.* (2011) 505:150–4. 10.1016/j.neulet.2011.10.009 22008503

[26] Maffei C Fossati A Agostoni I Barraco A Bagnato M Deborah D Interrater reliability and internal consistency of the structured clinical interview for DSM-IV axis II personality disorders (SCID-II), Version 2.0. *J Pers Disord.* (1997) 11:279–84. 10.1521/pedi.1997.11.3.279 9348491

[27] Hamilton M. A rating scale for depression. *J Neurol Neurosurg Psychiatry.* (1960) 23:56–62. 10.1136/jnnp.23.1.56 14399272 PMC495331

[28] Young RC Biggs JT Ziegler VE Meyer DA. A rating scale for mania: reliability, validity and sensitivity. *Br J Psychiatry.* (1978) 133:429–35. 10.1192/bjp.133.5.429 728692

[29] Hamilton M. The assessment of anxiety states by rating. *Br J Med Psychol.* (1959) 32:50–5. 10.1111/j.2044-8341.1959.tb00467.x 13638508

[30] Woerner MG Mannuzza S Kane JM. Anchoring the BPRS: an aid to improved reliability. *Psychopharmacol Bull.* (1988) 24:112–7. 3387514

[31] Yang J Ouyang X Tao H Pu W Fan Z Zeng C Connectomic signatures of working memory deficits in depression, mania, and euthymic states of bipolar disorder. *J Affect Disord.* (2020) 274:190–8. 10.1016/j.jad.2020.05.058 32469803

[32] Fan Z Yang J Zeng C Xi C Wu G Guo S Bipolar mood state reflected in functional connectivity of the hate circuit: a resting-state functional magnetic resonance imaging study. *Front Psychiatry.* (2020) 11:556126. 10.3389/fpsyt.2020.556126 33192670 PMC7652934

[33] Wechsler D. *Wechsler Adult Intelligence Scale–Fourth Edition (WAIS–IV).* San Antonio, TX: Pearson (2008).

[34] Yan CG Wang XD Zuo XN Zang YF. DPABI: data processing & analysis for (resting-state) brain imaging. *Neuroinformatics.* (2016) 14:339–51. 10.1007/s12021-016-9299-4 27075850

[35] Bandettini PA Jesmanowicz A Van Kylen J Birn RM Hyde JS. Functional MRI of brain activation induced by scanner acoustic noise. *Magn Reson Med.* (1998) 39:410–6. 10.1002/mrm.1910390311 9498597

[36] Yang J Pu W Ouyang X Tao H Chen X Huang X Abnormal connectivity within anterior cortical midline structures in bipolar disorder: evidence from integrated MRI and functional MRI. *Front Psychiatry.* (2019) 10:788. 10.3389/fpsyt.2019.00788 31736805 PMC6829675

[37] Zuo XN Ehmke R Mennes M Imperati D Castellanos FX Sporns O Network centrality in the human functional connectome. *Cereb Cortex.* (2012) 22:1862–75. 10.1093/cercor/bhr269 21968567

[38] Leonardi N Van De Ville D. On spurious and real fluctuations of dynamic functional connectivity during rest. *Neuroimage.* (2015) 104:430–6. 10.1016/j.neuroimage.2014.09.007 25234118

[39] Leucht S Samara M Heres S Patel MX Woods SW Davis JM. Dose equivalents for second-generation antipsychotics: the minimum effective dose method. *Schizophr Bull.* (2014) 40:314–26. 10.1093/schbul/sbu001 24493852 PMC3932104

[40] Geng X Xu J Liu B Shi Y. Multivariate classification of major depressive disorder using the effective connectivity and functional connectivity. *Front Neurosci.* (2018) 12:38. 10.3389/fnins.2018.00038 29515348 PMC5825897

[41] Qiu M Zhang H Mellor D Shi J Wu C Huang Y Aberrant neural activity in patients with bipolar depressive disorder distinguishing to the unipolar depressive disorder: a resting-state functional magnetic resonance imaging study. *Front Psychiatry.* (2018) 9:238. 10.3389/fpsyt.2018.00238 29922189 PMC5996277

[42] Zhang Z Bo Q Li F Zhao L Wang Y Liu R Increased ALFF and functional connectivity of the right striatum in bipolar disorder patients. *Prog Neuropsychopharmacol Biol Psychiatry.* (2021) 111:110140. 10.1016/j.pnpbp.2020.110140 33068681

[43] Luo Z Chen G Jia Y Zhong S Gong J Chen F Shared and specific dynamics of brain segregation and integration in bipolar disorder and major depressive disorder: a resting-state functional magnetic resonance imaging study. *J Affect Disord.* (2021) 280:279–86. 10.1016/j.jad.2020.11.012 33221713

[44] Wang Y Gao Y Tang S Lu L Zhang L Bu X Large-scale network dysfunction in the acute state compared to the remitted state of bipolar disorder: a meta-analysis of resting-state functional connectivity. *Ebiomedicine.* (2020) 54:102742. 10.1016/j.ebiom.2020.102742 32259712 PMC7136605

[45] Fransson P Marrelec G. The precuneus/posterior cingulate cortex plays a pivotal role in the default mode network: evidence from a partial correlation network analysis. *Neuroimage.* (2008) 42:1178–84. 10.1016/j.neuroimage.2008.05.059 18598773

[46] Magioncalda P Martino M Conio B Escelsior A Piaggio N Presta A Functional connectivity and neuronal variability of resting state activity in bipolar disorder–reduction and decoupling in anterior cortical midline structures. *Hum Brain Mapp.* (2015) 36:666–82. 10.1002/hbm.22655 25307723 PMC6869107

[47] Zhong S Chen G Zhao L Jia Y Chen F Qi Z Correlation between intrinsic brain activity and thyroid-stimulating hormone level in unmedicated bipolar II depression. *Neuroendocrinology.* (2019) 108:232–43. 10.1159/000497182 30673659

[48] Martino M Magioncalda P Saiote C Conio B Escelsior A Rocchi G Abnormal functional-structural cingulum connectivity in mania: combined functional magnetic resonance imaging-diffusion tensor imaging investigation in different phases of bipolar disorder. *Acta Psychiatr Scand.* (2016) 134:339–49. 10.1111/acps.12596 27273612

