# Supplementary material for: Shared and distinct patterns of dynamical degree centrality in bipolar disorder across different mood states
Source: Front Psychiatry. 2022 Jul 27;13:941073. doi: 10.3389/fpsyt.2022.941073 (PMC9364672; doi:10.3389/fpsyt.2022.941073)
Supplement: Supplementary file 1 [file Data_Sheet_1.pdf]

Table S1. Details information of clinical scales scores across all patient groups.

Characteristics	BD (n= 28)	BM (n=13)	BE (n= 21)	*F/χ2*	*P*
HAMD	21.68±3.86	4.38±4.073	5.19±4.07	122.500	<0.001[b]
HAMA	16.52±8.03	2.69±4.19	4.24±3.96	33.129	<0.001[b]
YMRS	1.37±1.64	21.92±7.01	3.43±2.91	140.063	<0.001[b]
BPRS	33.20±8.17	26.77±6.91	22.80±3.79	13.874	<0.001[b]

Note: [b]*P* values for one-way ANOVA. Values are presented by mean ± standard deviation. **Abbreviation:** BD, bipolar depression; BM, bipolar mania; BE, bipolar euthymia; HCs, healthy controls; WAIS, Wechsler Intelligence Scale; HAMD, 17-item Hamilton Depression Rating Scale; HAMA, Hamilton Anxiety Rating Scale; YMRS, Young Mania Rating Scale; BPRS, Brief Psychiatric Rating Scale; N/A, not available.

Figure S1. Correlation analyses between the dDC variability of areas with omnibus differences and mean FD in all participants.

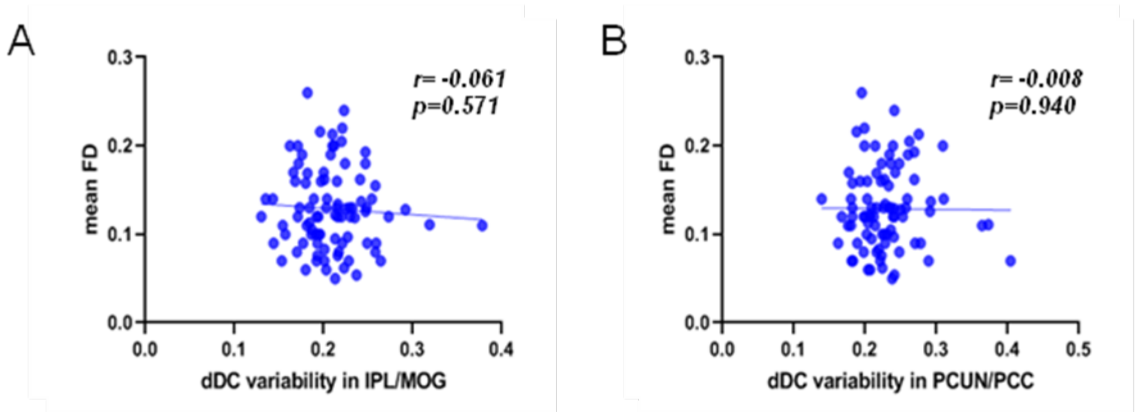

Abbreviations: dDC, the dynamical degree centrality; FD, framewise displacement; IPL/MOG, inferior parietal lobule/middle occipital gyrus; PCUN/PCC, precuneus/posterior cingulate cortex.

Table S2. Brain regions with significant dDC difference among the four groups by using 70 TRs (140 s) as sliding window size.

One-way ANOVA							Post-hoc analysis	
**Brain region**	**MNI**			**BA**	**Voxels**	***F***	**Comparisons**	***T***
	**X**	**Y**	**Z**					
IPL/MOG	-42	-57	54	40	37	11.02	BD<BM	-3.69
							BD<BE	-4.82
							BD<HCs	-5.24
PCUN/PCC	9	-48	15	30	23	6.39	BD<BE	-3.38
							BD<HCs	-3.21
							BM<BE	-2.23

Abbreviations: dDC, the dynamical degree centrality; BD, bipolar depression; BM, bipolar mania; BE, bipolar euthymia; HCs, healthy controls; MNI, Montreal Neurological Institute, BA, Brodmann area. IPL/MOG, inferior parietal lobule/middle occipital gyrus; PCUN/PCC, precuneus/posterior cingulate cortex.
